# Supplementary material for: Robot-enhanced diabetes care for middle-aged and older adults living with diabetes in the community: A small sample size mixed-method evaluation
Source: PLoS One. 2022 Apr 15;17(4):e0265384. doi: 10.1371/journal.pone.0265384 (PMC9012373; doi:10.1371/journal.pone.0265384)
Supplement: Attachment — Submitted filename: Response to Reviewers.docx [file pone.0265384.s001.docx]

December 13, 2021

Mieke Van Hemelrijck

Editor-in-Chief

*PLoS One*

Dear Dr. Hemelrijck:

I appreciate the thorough and expert review of our manuscript entitled “Robot-enhanced diabetes care for middle-aged and older adults living with diabetes in the community: a mixed-method evaluation.” I am encouraged by the positive responses to this work and believe I have addressed all criticisms and suggestions. Each of the reviewers’ concerns offers me the opportunity to clarify this study and strengthen the findings. I have enclosed the responses to the reviewers’ comments, along with page numbers indicating where the changes can be found in the revised manuscript.

Technological development in the field of healthcare is progressing rapidly. However, the actual needs of patients and the results of medical care using technology are rarely calibrated. In the present study, a mixed method evaluation was employed to collect quantitative and qualitative data on diabetes patients and community pharmacists related to a newly-invented diabetes-care robot. Instead of testing it in the laboratory, we tested it at a community pharmacy through interactions with patients in the community. Findings from the present study revealed the impact of the robot from different perspectives including the diabetes patients and the pharmacist. We believe that the findings of the present study suggest the potential for sustainable development of technology in the diabetes care field.

Again, we offer our thanks to you and the anonymous reviewers for your feedback. We look forward to a favorable reply.

Sincerely,

Lin-Chun Hua

Institute of Gerontology, College of Medicine, National Cheng Kung University, No. 1, University Road, Tainan, Taiwan, 70101.

Tel: +886-6-2353535 ext. 5977 (voice), +886-6-3028175 (fax)

E-mail: flower921flora@gmail.com

**Manuscript: “Robot-enhanced diabetes care for middle-aged and older adults living with diabetes in the community: a mixed-method evaluation” [PONE-D-21-35505] - [EMID:36edb3f2705449f1]**

**Reviewer #** **1:**

1. *Firstly, I would like to thank the editor for letting me review this manuscript. It was an interesting one and would congratulate the authors for thinking in the direction of spreading robotic-based diabetes awareness for the elderly and middle-aged people. It must be a difficult task, planning, setting-up, and implementing the study. As they have highlighted using the robotic approach significantly improved diabetes-related knowledge amongst the patients with diabetes, such studies on a larger scale must be conducted to see the actual effect. Although it's difficult but with collaboration and funding other regions and people with different socio-economic backgrounds could be included.*

**AUTHOR’S RESPONSE:** The authors appreciate the reviewer’s positive evaluation of our work. Due to the instability of the prototype robot, it is difficult to carry out a larger scale research currently. But we added this important point to the conclusion as expectations in the future research (Line 1002, page 63).

2. *The data is well-presented and analyses also seems to be extensively done. Although the authors have mentioned in the limitations about small sample size, I think they can consider adding it in the "Methods" section or "Title" perhaps.*

**AUTHOR’S RESPONSE:** The authors appreciate this important advice. A note on this will be added to the methods (Line 114 and line 115, page 7). The sentences were marked with blue. Thank you for the title suggested. The precedent version of the title has been replaced, becoming “Robot-enhanced diabetes care for middle-aged and older adults living with diabetes in the community: a small sample size mixed-method evaluation”.

3. *In the "Measures" section...please cross-check if there is repetition...Line 165 and line 168.*

**AUTHOR’S RESPONSE:** The authors appreciate the reviewer for pointing out this problem. Thank you for the detailed review. We have removed the repetitive sentences and marked the removed ones with yellow.

**Reviewer #** **2:**

1. *Great effort, congratulations to authors on this detailed manuscript. I don't have any significant comments, but since I am a physician, I find it very tiring text to read and I recommend to shorten the manuscript much further to allow for larger audience of readers to benefit from this paper.*

**AUTHOR’S RESPONSE:** The authors appreciate this important suggestion. We have significantly shortened our manuscript from 67 to 62 pages.

2. *I also would like to ask to include information in the abstract about the population studied in this paper who are middle age and older population. As I read the abstract first, I didn't release you were targeting primary a specific age group and it would be optimal if that information is included in the abstract.*

**AUTHOR’S RESPONSE:** The authors appreciate this important advice. We have added the age of the diabetes patients in the abstract.
